# How should we interpret excessive left ventricular trabeculation? Update on controversies from the cardiac imaging perspective

**DOI:** 10.1186/s13244-026-02264-x

**Published:** 2026-05-14

**Authors:** Roque Oca Pernas, Nerea Hormaza Aguirre, Marcos Mestas Nuñez, Ana Capelastegui Alber, María Navallas Irujo, Ana García Durán, Alex Pérez Casares, Flavio Zuccarino

**Affiliations:** 1 MR Department, OSATEK Deusto, Osakidetza-Bask Health Service, Bilbao, Spain; 2 Radiology Department, Cruces University Hospital, Biocruces Bizkaia, Barakaldo, Spain; 3 Radiology Department, Hospital del Mar University Hospital, Barcelona, Spain; 4 MR Department, OSATEK Galdakao University Hospital, Osakidetza-Bask Health Service, Galdakao, Spain; 5 Radiology Department, Sant Joan de Deu University Hospital, Barcelona, Spain; 6 Cardiology Department, Hospital del Mar University Hospital, Barcelona, Spain; 7 Cardiology Department, Sant Joan de Deu University Hospital, Barcelona, Spain

**Keywords:** Magnetic resonance imaging, Heart ventricles, Myocardium, Myocardial trabeculation, Left ventricular noncompaction

## Abstract

**Abstract:**

There are well-established and widely accepted criteria for determining the presence of excessive myocardial trabeculation (ET) in the left ventricle in patients undergoing cardiac imaging studies. ET has been documented in healthy individuals, as well as in patients with cardiomyopathies. It is also associated with clinical conditions that increase preload and afterload, as well as various neuromuscular and systemic diseases. There is sufficient scientific evidence demonstrating that the development of ET is not due to an embryologic interruption in myocardial compaction. Therefore, the term “ventricular non-compaction” is now outdated, and its classification as an independent cardiomyopathy is discouraged. However, significant controversy remains regarding the clinical relevance of this phenotypic trait and its implications for the management of patients with suspected or diagnosed cardiovascular disease. This review aims to provide a comprehensive and updated overview of current knowledge on myocardial trabeculation, including diagnostic criteria, prognostic implications, and its associations with other conditions, with a particular focus on differences between adult and pediatric populations. Furthermore, it discusses the potential adverse cardiovascular events linked to ET and highlights the importance of differential diagnosis to distinguish myocardial ET from other mimicking conditions.

**Critical relevance statement:**

This review critically appraises current knowledge on myocardial trabeculation, integrating imaging and clinical perspectives, to clarify diagnostic criteria, highlight differential diagnoses, and improve diagnostic accuracy and clinical decision-making.

**Key Points:**

- ET does not necessarily represent a pathological imaging finding and may be related to a normal phenotypic trait.
- Hemodynamic stressors may trigger excessive trabeculation, though its cause and clinical significance remain unclear.
- Excessive trabeculation imaging must combine with clinical and genetic information for accurate prognostic stratification.

**Graphical Abstract:**

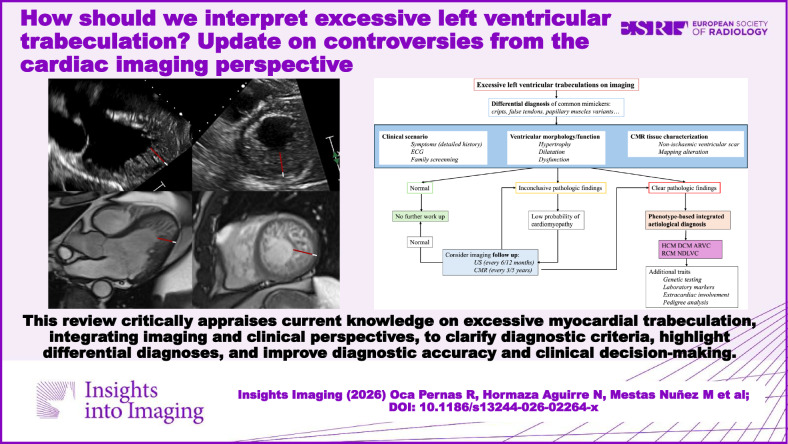

## Introduction

The left ventricular (LV) wall is composed of two structurally distinct layers: the inner trabeculated layer and the outer compact layer. The ratio between the

thickness of both layers is not constant and can be accurately identified using non-invasive cardiac imaging techniques, primarily echocardiography (US) and cardiac magnetic resonance imaging (CMR) [1, 2]. Traditionally, the disproportionate development of the trabeculated layer was thought to result from a failure in embryonic myocardial compaction, potentially leading to clinical manifestations and being classified as a rare form of non-compacted cardiomyopathy, which could additionally trigger major cardiac events [3–5].

Although many controversies regarding myocardial trabeculation remain, it is now known that excessive trabeculation (ET) is not secondary to an arrest in myocardial compaction, and it is not necessarily a pathological trait in asymptomatic individuals [6–8]. However, myocardial trabeculae still might contribute to the pathophysiology of ventricular remodeling, and it is likely that their development is influenced by genetic mechanisms of an underlying cardiomyopathy [9, 10].

Although the terms ‘hypertrabeculation’ and ‘ET’ have been used interchangeably in the literature, in the present review, the term ET will be used consistently to ensure terminological clarity and uniformity.

This article aims to summarize the advances in knowledge on ET over the past years from a cardiac imaging perspective. It explains the embryological pathogenetic and diagnostic criteria of ET, the new approach to this phenotypic trait within cardiomyopathy guidelines, and its relationship with other diseases or conditions that increase preload or afterload. Additionally, it outlines the differences in trabeculation between adults and the pediatric population, addresses the cardiovascular complications that have traditionally been considered consequences of left ventricular “non-compaction” (LVNC), and provides an iconographic guide highlighting the main differential diagnoses of ET.

## Embryological concepts

In vertebrates, cardiac development follows a common initial pattern from a primordial embryonic tube, which subsequently differentiates into the cardiac chambers under the control of a complex network of genetic regulatory mechanisms. During the early embryonic period, these cardiac chambers exhibit a trabeculated endoluminal surface that confers the ventricles a spongiform appearance. The trabecular surface enables nutrient and oxygen diffusion in early development and is crucial for the development of the conduction system and ventricular chamber maturation [11–13].

In ectothermic animals, this trabeculated structure persists throughout adulthood, whereas in endothermic animals (birds and mammals), fully developed ventricles present a well-defined compact layer [14, 15].

Nevertheless, no biomechanical benefits have been identified from the presence of compact myocardium. In fact, cold-blooded animals, with trabeculated ventricles, demonstrate higher ejection fractions and cardiac output, higher heart rates, and higher blood pressures compared to endothermic animals with compact myocardium [16, 17]. Although the cardiomyocytes of both inner and outer layers have a similar structure, their response to different physiological or pathological scenarios may not be the same.

The development of the compact layer of the human heart increases significantly from the seventh week of gestation, in a non-proportional manner relative to the trabeculated myocardium, which grows more slowly; this is known as an allometric growth pattern. Different immunohistochemical models have demonstrated that the formation of the compact layer does not occur at the expense of a reduction in the trabeculated layer of the myocardium; rather, both layers develop independently [18, 19].

For all the above reasons, the hypothesis of intrauterine arrest of myocardial compaction at the expense of the trabeculated layer to justify the presence of ET myocardium appears to be outdated.

## Genetic determinants

A diverse array of genes encoding sarcomeric, cytoskeletal, mitochondrial, or ion channel proteins has been associated with a phenotype characterized by ET (e.g., MYH7, MIB1, DES, MYBPC3, TTN, ACTN2, LMNA, PLN, TBX20, TBX5, DTNA, and TNNT2) (9,20).

More than 18 distinct loci for chromosomal abnormalities have been reported in association with so-called LVNC. Since many of these abnormalities often affect multiple genes and intronic regions that may have regulatory functions, their precise impact remains unclear. Around 30% of patients with ET have a recognized genetic variant associated with the phenotype. Of these, approximately one-third carry variants in genes encoding sarcomeric proteins. Most genetic causes are missense mutations (55%), with autosomal dominant inheritance being the most common pattern (83%), whereas X-linked and mitochondrial inheritance are less frequent [20–24].

Trabeculation-associated loci may function as risk factors or disease modifiers in cardiomyopathy, with their effects varying based on the genetic background. Moreover, increased trabeculation is a feature of both hypertrophic and dilated cardiomyopathy (DCM); however, in association with certain genes, the phenotype may predominantly manifest as isolated ET. In addition, many genetic variants linked to myocardial trabeculation exhibit opposing effects depending on the type of underlying cardiomyopathy; for instance, pathogenic ACTC1 mutations can lead to ventricular dilatation or hypertrophy with ET, contingent upon the underlying cardiomyopathy [21, 23]. The main genes associated with ET are summarized in Table 1.

**Table 1:** Summary of prominent genes associated with ETs

Gene	Association degree with ET	Associations with other conditions
*ACTC1*	Definitive	HCM (definitive), DCM (moderate), ARVC (no known relationship)
*ACTN2*	Limited	Intrinsic cardiomyopathy (moderate: missense variants only)
*ALPK3*	Moderate	HCM (definitive)
*DES*	Definitive	DCM (definitive), ARVC (moderate)
*DMD*	Moderate	Progressive muscular dystrophy (definitive)
*DMPK*	Moderate	Myotonic dystrophy 1
*DSP*	Definitive	ARVC (definitive), DCM (strong), HCM (disputed)
*HCN4*	Moderate	Familial thoracic aortic aneurysm and aortic dissection (limited), Brugada syndrome (limited)
*LAMP2*	Moderate	Danon disease (definitive)
*LDB3*	Moderate	DCM (limited), ARVC (disputed)
*LMNA*	Moderate	DCM (definitive), ARVC (limited)
*MIB1*	Definitive	DCM (no known relationship)
*MYBPC3*	Definitive	HCM (definitive), DCM (limited), ARVC (limited)
*MYH7*	Definitive	HCM (definitive), DCM (definitive), ARVC (limited)
*MYL2*	Moderate	HCM (definitive), DCM (limited), ARVC (no known relationship)
*NEXN*	Moderate	DCM (moderate), HCM (limited)
*NKX2-5*	Moderate	NKX2.5-related congenital, conduction, and myopathic heart disease (definitive), DCM (limited)
*NNT*	Moderate	Glucocorticoid deficiency 4 (definitive)
*NONO*	Definitive	X-linked syndromic intellectual disability (definitive)
*PKP2*	Moderate	ARVC (definitive), DCM (disputed)
*PLN*	Moderate	Intrinsic cardiomyopathy (definitive), ARVC (moderate)
*PRDM16*	Moderate	DCM (limited)
*RBM20*	Moderate	DCM (definitive)
*RYR2*	Definitive	CPVT (definitive), HCM (limited), ARVC (refuted)
*SCN5A*	Moderate	DCM (definitive), ARVC (disputed), Brugada syndrome (definitive), LQTS (definitive)
*TAFAZZIN*	Definitive	Barth syndrome (definitive)
*TBX5*	Moderate	Holt-Oram syndrome (definitive)
*TBX20*	Moderate	DCM (limited)
*TMEM70*	Moderate	Mitochondrial disease (definitive)
*TNNC1*	Moderate	DCM (definitive), HCM (moderate), ARVC (no known relationship)
*TNNI3*	Moderate	HCM (definitive), DCM (moderate), ARVC (no known relationship)
*TNNT2*	Moderate	HCM (definitive), DCM (definitive), ARVC (no known relationship)
*TPM1*	Definitive	HCM (definitive), DCM (moderate), ARVC (no known relationship)
*TTN*	Definitive	DCM (definitive), HCM (limited), ARVC (limited)
*VCL*	Moderate	DCM (moderate), HCM (limited)

Adapted from ref. [10]

*ARVC* arrhythmogenic right ventricular cardiomyopathy, *CPVT* catecholaminergic polymorphic ventricular tachycardia, *DCM* dilated cardiomyopathy, *ET* excessive trabeculations, *HCM* hypertrophic cardiomyopathy, *LQTS* long QT syndrome

For these reasons, genetic testing should not rely exclusively on the presence of ET in asymptomatic patients with otherwise normal cardiac findings, but should be recommended based on the presence of features consistent with a conventional cardiomyopathy [25, 26].

## Imaging findings

The diagnosis of myocardial ET is based on imaging techniques, which allow for the detection of a trabeculated inner layer with deep intertrabecular recesses, thicker than the outer layer of compact myocardium [27].

US represents the first-line imaging test, mainly due to its high availability and favorable cost-effectiveness ratio [28]. However, the technique has general limitations, including its operator-dependent nature and potential challenges in visualizing the ventricular apex [29]. Furthermore, the US may struggle to clearly differentiate between the trabeculated and the compact layer, particularly in the end-diastolic phase; and short-axis plane alignment may not always be strictly perpendicular, leading to errors in ventricular wall thickness quantification [28, 29].

The most widely used echocardiographic criteria are those established by Jenni et al, which define ET when the ratio of trabeculated to compact myocardium thickness exceeds 2, measured in parasternal short-axis views during end-systole [30].

In addition to the Jenni criteria, Stöllberger et al proposed echocardiographic diagnostic criteria in 2002, defining ET when more than three prominent trabeculations are visible in one imaging plane, apically to the papillary muscles, moving synchronously with the myocardium and perfused from the ventricular cavity, with intertrabecular spaces visualized by color Doppler [31]. In 2013, the same group refined these criteria to enhance diagnostic accuracy, emphasizing that both end-systolic and end-diastolic images should be evaluated and that the simultaneous presence of more than three trabeculations and a clearly two-layered myocardial structure are required for diagnosis. They also recommended the use of atypical imaging planes when standard views are inconclusive and advised assessing the ratio of trabeculated to compacted myocardium in end-diastole, particularly in apical four-chamber views, to avoid overdiagnosis [32].

**Fig. 1:**
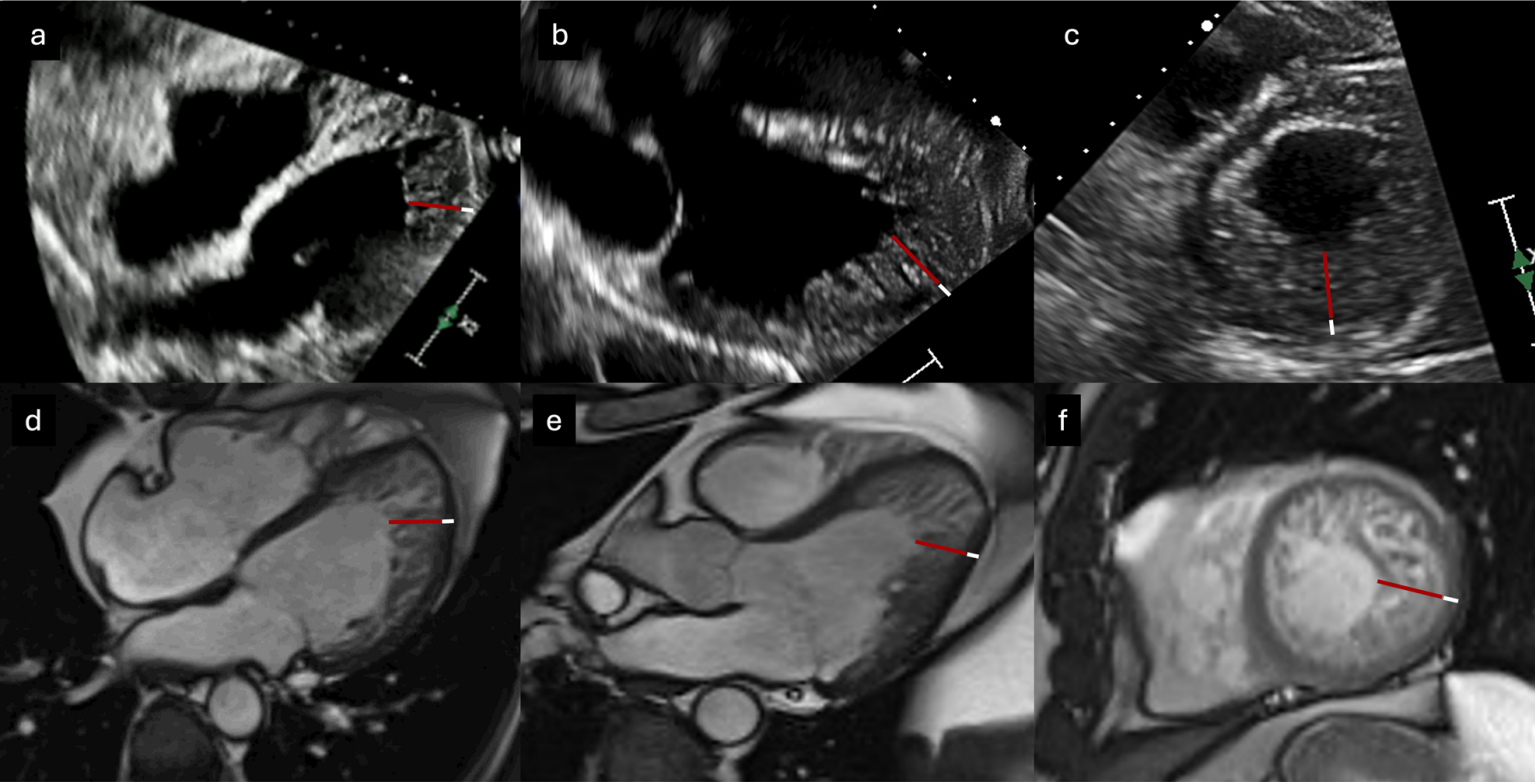
US and CMR diagnosis of ET. **a** US four-chamber view in end-systole; (**b**) US three-chamber view in end-systole, (**c**) US apical short-axis view in end-systole; **d** CMR steady-state free precession [SSFP] cine four-chamber view in end-diastole; **e** CMR SSFP cine three-chamber view in end-diastole; and (**f**) CMR SSFP cine apical short-axis view in end-diastole) show prominent LV trabeculations and deep intertrabecular recesses (red line) relative to the compact myocardial layer (white line)

CMR is increasingly employed for diagnosing patients with cardiovascular disease, primarily due to its multiplanar imaging capability with wide fields of view, absence of ionizing radiation, and combination of high spatial and contrast resolution, allowing for highly precise cardiac tissue characterization. Thanks to improved contrast between the ventricular cavity and trabeculae compared to US, it enables a reliable diagnosis of ET, determining its distribution and extent [33]. Representative echocardiographic and CMR images illustrating these morphological features are shown in Fig. 1.

Therefore, CMR is commonly used to confirm the diagnosis of myocardial ET, especially valuable when echocardiographic image quality is suboptimal, and to evaluate for additional signs of cardiomyopathy or congenital heart disease [28]. CMR sequences used for the assessment of myocardial trabeculations are summarized in Table 2.

**Table 2:** CMR sequences for the assessment of myocardial trabeculations

MRI sequences	Standard imaging planes employed	Main utilities
Cine SSFP	Long axis (3C, 4C, 2C), SA stack	Morphological assessment: wall thickness and myocardial mass quantification, trabeculae measurements. Functional evaluation: global and regional contraction assessment; volume and function quantification. Valves evaluation
T1, T2, T2^*^ mapping	4C and mid-ventricular SA	Detection and quantification of myocardial fibrosis, edema, fat, iron, and ECV
Perfusion imaging	Basal, medium, and apical SA	Detection and quantification of myocardial ischemia
Phase contrast	Through-plane and in-plane 2D, and 4D	Detection and quantification of valvular pathology and shunts
LGE	Planning equivalent to cine sequences	Detection and quantification of myocardial fibrosis, infarct, infiltrative disease, and pericardial and myocardial inflammation
Strain	Postprocessing analysis derived from cine SSFP sequences	Regional myocardial function evaluation. Early myocardial dysfunction assessment

*2C* two-chamber, *3C* three-chamber, *4C* four-chamber, *2D* two-dimensional, *3D* three-dimensional, *4D* four-dimensional, *ECV* extracellular volume, *Gd* gadolinium, *GRE* gradient recall echo, *LGE* late gadolinium enhancement, *SA* short axis, *SSFP* steady-state free precession

CMR may reveal key etiological information and should be interpreted alongside genetic testing and clinical findings. Serial follow-up with CMR may be useful in case of monitoring treatment response and disease evolution is needed, typically every 2–5 years, depending on initial severity and disease progression [34].

The evaluation of ET strongly depends on MR image quality, particularly in bright-blood cine imaging. Spatial resolution is critical, as limited resolution may lead to inaccurate assessment of trabecular thickness. Motion artifacts from respiration, suboptimal gating, and cardiac arrhythmias can further degrade image sharpness and obscure fine endocardial details. In addition, the increasing use of accelerated acquisitions, such as compressed sensing, although highly valuable in reducing scan time, may introduce reconstruction artifacts, such as residual aliasing or noise amplification, or the loss of subtle trabecular structures that are diagnostically relevant. These limitations underscore the importance of careful protocol optimization and standardization across centers, as variability in cine image quality may contribute to both over- and underdiagnosis of ET [2].

The use of contrast-enhanced CMR is considered a Class I recommendation for the initial diagnosis of patients with suspected cardiomyopathy, and a Class IIa recommendation for disease monitoring and follow-up [34].

The CMR Petersen criteria are the most widely accepted and define ET as a ratio of trabeculated to compacted myocardium greater than 2.3, measured in end-diastole in three long-axis cine views (two-chamber, four-chamber, and LV outflow tract views). The maximal ratio obtained among these three views is used for analysis. In addition, and as stated in the original publication, the apical segment (segment 17) was excluded from measurements because the compacted myocardium is generally thinner in this area, and inclusion would have resulted in artificially high ratios [35]. Other approaches have also been utilized, including those that consider volume of trabeculation, such as Jacquier’s criteria, which set a cutoff of 20% trabeculated myocardial mass as the higher limit of normality [36]. Additionally, some methods have analyzed the geometric distribution of trabeculae based on their fractal dimension, in an attempt to reflect the complexity of the trabeculae and allow for a more accurate three-dimensional assessment [37]. A summary of the main imaging criteria used to define and quantify ET is provided in Table 3.

**Table 3:** Imaging criteria for determining the diagnosis and extent of LV ET

Criteria	Modality	Definition	Cardiac phase	Cardiac plane	Positive diagnosis of ET
Petersen et al	CMR	Two-layered myocardium appearance. Measured at the most pronounced trabeculations, avoiding apex. Measurement perpendicular to Compact myocardium	End-diastole	3 Long axis (two-chamber, four-chamber, and LV outflow tract views)	Noncompaction to compaction myocardium thickness ratio > 2.3
Jacquier et al	CMR	Short-axis cines for total LV mass and compact mass to define trabecular mass The papillary muscle is included in the myocardial mass.	End-diastole	Short-axis stack	Trabecular mass > 20%
Captur et al	CMR	Loss of base-to-apex fractional dimension gradient	End-diastole	Short-axis stack	Fractal dimension ≥ 1.30
Jenni et al	US	Noncompaction to compaction ratio. Decreased thickening and hypokinesia are present in noncompacted segments	End-systole	Short axis	Noncompaction to compaction ratio > 2
Stöllberger et al (initial criteria)	US	More than three prominent trabeculations apically to the papillary muscles, moving synchronously with the myocardium and with intertrabecular spaces visualized by color Doppler	-	Four-chamber view	> 3 trabeculations protruding from the apical LV wall
Stöllberger et al (refined criteria)	US	More than three trabeculations and a clearly two-layered myocardial structure. These trabeculations moved synchronously with the compacted myocardium.	End-systole and end-diastole	Four-chamber view and additional atypical imaging planes if necessary	- > 3 prominent trabeculations - Two-layered myocardial structure - Perfusion of the intertrabecular spaces on color-Doppler US or contrast US
Grothoff et al	CMR	LV epicardial borders were manually traced in end-systole and end-diastole	End-systole and end-diastole	Four-chamber view and vertical long-axis view orientation	Trabeculated ventricular mass greater than 25% of the global LV mass; noncompacted mass greater than 15 g/m^2^

*CMR* cardiac MR imaging, *LV* left ventricle, *US* echocardiography

Recently, artificial intelligence algorithms have been developed to fully automate the segmentation and quantification of trabeculation and papillary muscles, demonstrating superior accuracy and reproducibility compared to human expert evaluations [38].

Regarding the right ventricle (RV), there are no well-defined morphological criteria to clearly establish the presence of ET. Physiologically, the RV is more trabeculated than the LV, making it difficult to distinguish between normal and ET [39]. As a result, this condition is likely underdiagnosed. A histological threshold has been proposed, suggesting that ET may be defined when trabeculated tissue exceeds 75% of the total wall thickness compared to compact myocardium [40]. When RV dilatation and dysfunction are also present, the possibility of an associated cardiomyopathy should be considered [41].

**Fig. 2:**
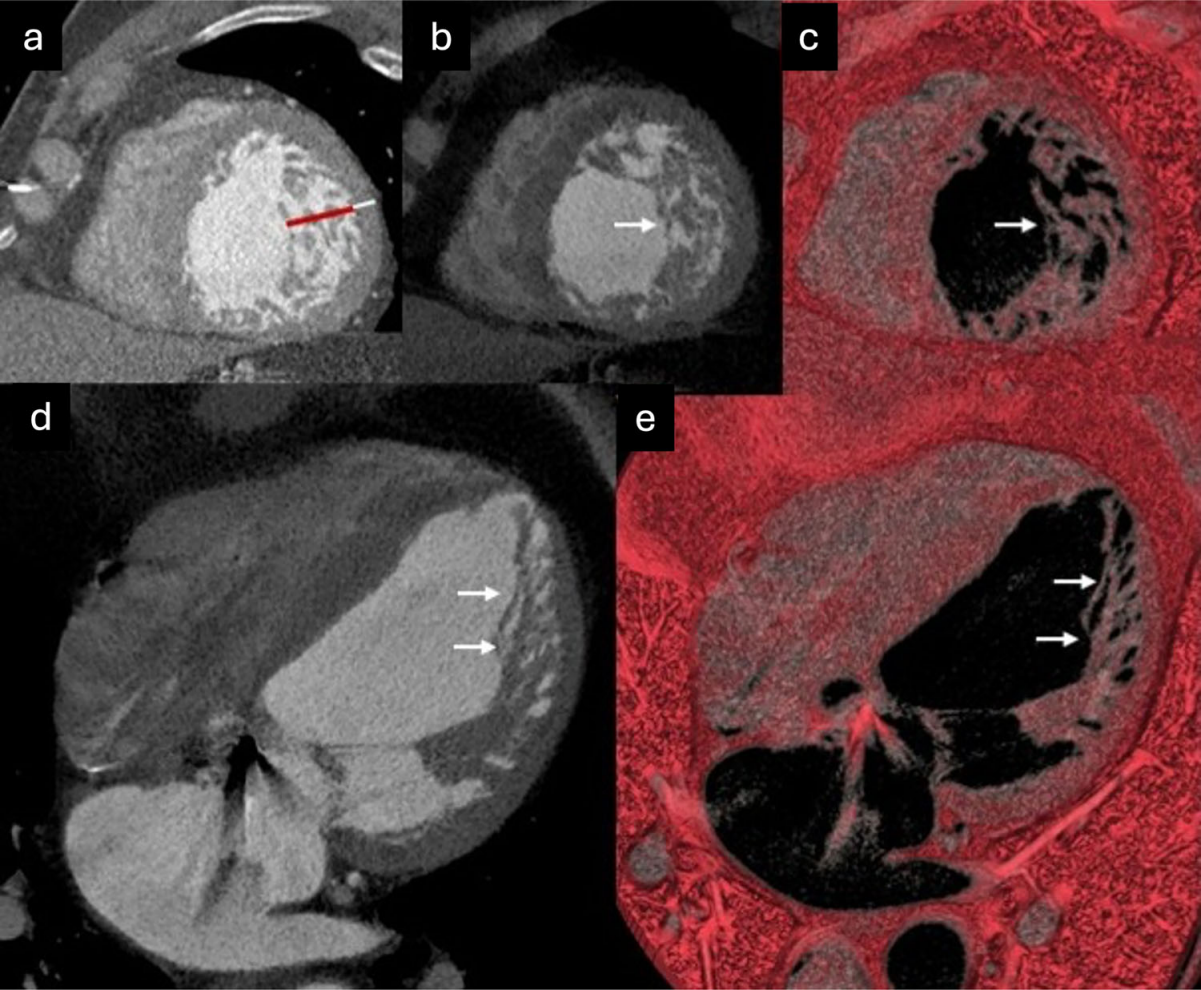
Cardiac CT performed to rule out coronary artery disease in a patient with chest pain incidentally demonstrates LV ET. **a** Multiplanar reconstruction [MPR] short-axis end-diastolic view showing the trabeculated layer [red line] and compact layer [white line]; (**b**, **d**) minimum intensity projection [MinIP] end-diastolic images; and (**c**, **e**) volume-rendered [VR] end-diastolic images) show a well-defined trabecular distribution, predominantly located in the lateral wall (arrows)

Multidetector CT, thanks to its high spatial resolution, enables precise identification of the two myocardial layers, facilitating accurate quantification of their thicknesses [42]. Due to the use of ionizing radiation in this technique, its lower tissue characterization capability, and its limited ability to detect fibrosis, its role in diagnosing ET is more restricted. However, the incorporation of the latest CT equipment, which provides acquisitions with very low radiation doses and spectral technology, could aid in the management of patients with suspected cardiomyopathy, particularly those in whom CMR is contraindicated or patients with congenital heart disease, suspected anomalies in coronary origin, or those requiring the exclusion of coronary artery disease [28, 42]. Cardiac CT examples of ET and the delineation of compact and trabeculated layers are presented in Fig. 2.

Although diagnostic criteria for ET using cardiac CT have been proposed, such as those by Meléndez-Ramírez et al, who defined ET as a trabeculated to compacted myocardium ratio > 2.2 measured in end-diastole in at least two LV segments [43], there is currently no standardized CT-based approach for reliably establishing the diagnosis. Nevertheless, several studies have explored CT-based criteria, highlighting both the potential and the limitations of this modality [44–46]. In clinical practice, prominent trabeculation may be incidentally detected on coronary CT angiography or other cardiac CT protocols. In such cases, the finding should be reported, and confirmatory assessment with CMR may be recommended when clinically indicated, particularly in the presence of diagnostic uncertainty or associated clinical suspicion [44]. CT acquisitions optimized for left-heart opacification may result in limited right ventricular enhancement, particularly in wide-detector systems or specific timing strategies. In this setting, accurate delineation of compact myocardium in septal segments may be challenging, potentially affecting trabeculated to compacted measurements. When septal assessment is limited, delayed or venous-phase acquisitions may improve myocardial conspicuity; however, CMR remains preferable for definitive evaluation [45].

## Clinical implications

The original criteria for diagnosing LVNC cardiomyopathy were based solely on the latter simplistic morphological parameters; and they were inconsistent, non-fully standardized, and based on limited patient series, leading to significant variability in diagnosis and contributing to LVNC overdiagnosis [47].

Moreover, these criteria did not incorporate functional parameters such as LV size, or systolic function, nor the presence of late gadolinium enhancement (LGE), which has likely further contributed to this overdiagnosis [6, 47].

The latest European Society of Cardiology (ESC) cardiomyopathy guidelines have removed LVNC as an independent entity, reclassifying ET as a phenotypic trait [34]. This Task Force advocates for a diagnostic approach centered on the predominant cardiac phenotype at presentation, incorporating both morphological characteristics (such as hypertrophy, dilation, and tissue characterization) and functional aspects (including systolic and diastolic ventricular dysfunction), to classify these morphofunctional characteristics within one of the five newly established cardiomyopathies.

LV ET is considered, therefore, a phenotypic trait that can be present in healthy individuals. In fact, studies based on CMR (including multi-ethnic and general population cohorts) have shown that between 15% and 43% of healthy individuals may meet imaging criteria for ET in at least one myocardial segment of the LV [48, 49]. It has been demonstrated that in asymptomatic individuals, the presence of ET does not imply a higher risk of ventricular remodeling, nor is this risk dependent on the extent or severity of the trabeculation [50].

Sex-related differences in myocardial trabeculation have also been reported. In population-based cohorts evaluated with CMR, women have been shown to exhibit slightly higher maximal non-compacted to compacted ratios than men, independent of age [48]. These differences should be considered when interpreting trabeculation patterns in clinical practice.

Ethnic differences in the extent of LV trabeculation have been described, although the evidence remains heterogeneous and method-dependent. In the multi-ethnic MESA cohort, no statistically significant difference in the mean trabeculated/compacted ratio was observed among White, African-American, Hispanic, and Chinese-American participants [50]. Conversely, smaller studies using fractal analysis have demonstrated greater trabecular complexity in healthy Black volunteers compared with White individuals [37]. Together, these findings also suggest that a higher degree of trabeculation may represent a physiological morphological variant in some ethnic groups, although interpretation should be cautious and consider the quantification method used.

ET has also been described as a physiological and reversible response to clinical conditions that increase the heart’s hemodynamic demands. This is especially observed in two characteristic groups: pregnant women and athletes [51–53].

The development of reversible ET in pregnant women is considered secondary to an increase in preload, which typically partially or completely resolves by 12 weeks postpartum. Ethnic differences have been observed among pregnant women in the degree of trabeculation, suggesting that the morphological response to increased hemodynamic demand is influenced by varying genetic susceptibility [54].

On the other hand, individuals who engage in intense physical activity have been found to develop ET more frequently than the general population. This is considered a morphological epiphenomenon that arises as a preload response to exercise, which varies depending on the type of sport and individual characteristics such as ethnicity and age [55, 56]. In asymptomatic athletes with ET, follow-up may not be required in the absence of a family history of cardiomyopathy, arrhythmia, or LV dysfunction; however, current EAPC guidelines emphasize an individualized approach [57].

Moreover, increased trabeculated LV mass has been recently associated, not only with greater physical activity levels, but also with individuals with hypertension, and elevated body mass index [58].

However, two clinical determinants have been described that may be associated with a worse prognosis and the development of major adverse cardiovascular events (MACE) in individuals with ET: ventricular dysfunction and the presence of myocardial fibrosis detected through LGE MRI sequences [59–62]. In these patients, the possibility of an underlying cardiomyopathy should be considered.

When ET is detected in the context of heart failure, another underlying cause (such as cardiomyopathy, arrhythmias, or congenital heart disease) is typically identified as the primary driver of the clinical presentation. This might also suggest that ET is an accompanying phenotypic trait rather than the causative substrate of the disease [10].

**Fig. 3:**
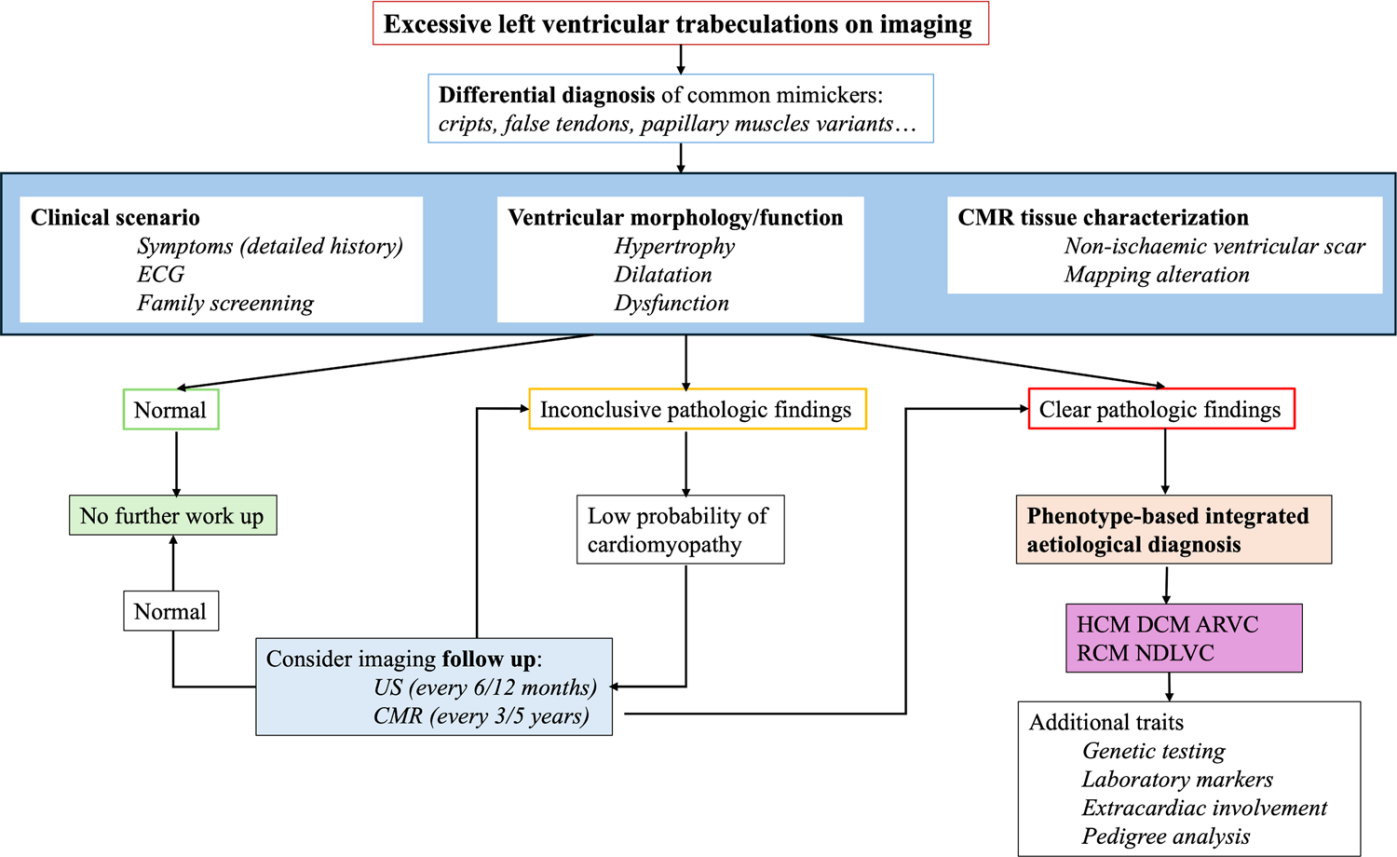
Diagnostic workflow of LV ETs in the adult population

In clinical practice, when no other findings are present beyond ET, there is still a risk stratification dilemma, as it is difficult to determine whether the individual is truly healthy and requires no further intervention or whether ET is due to a subclinical cardiomyopathy that could benefit from closer monitoring. A holistic approach is recommended in these cases, integrating all clinical information, functional and imaging tests, as well as family screening and genetic testing, to improve their diagnostic assessment [34, 63, 64]. The proposed diagnostic workflow for patients with ET is summarized in Fig. 3. This algorithm is intended to serve as a flexible guide, acknowledging that optimal follow-up intervals and imaging strategies should be tailored to the individual clinical context.

## Neuromuscular disease and other systemic disorders associated with ET

**Fig. 4:**
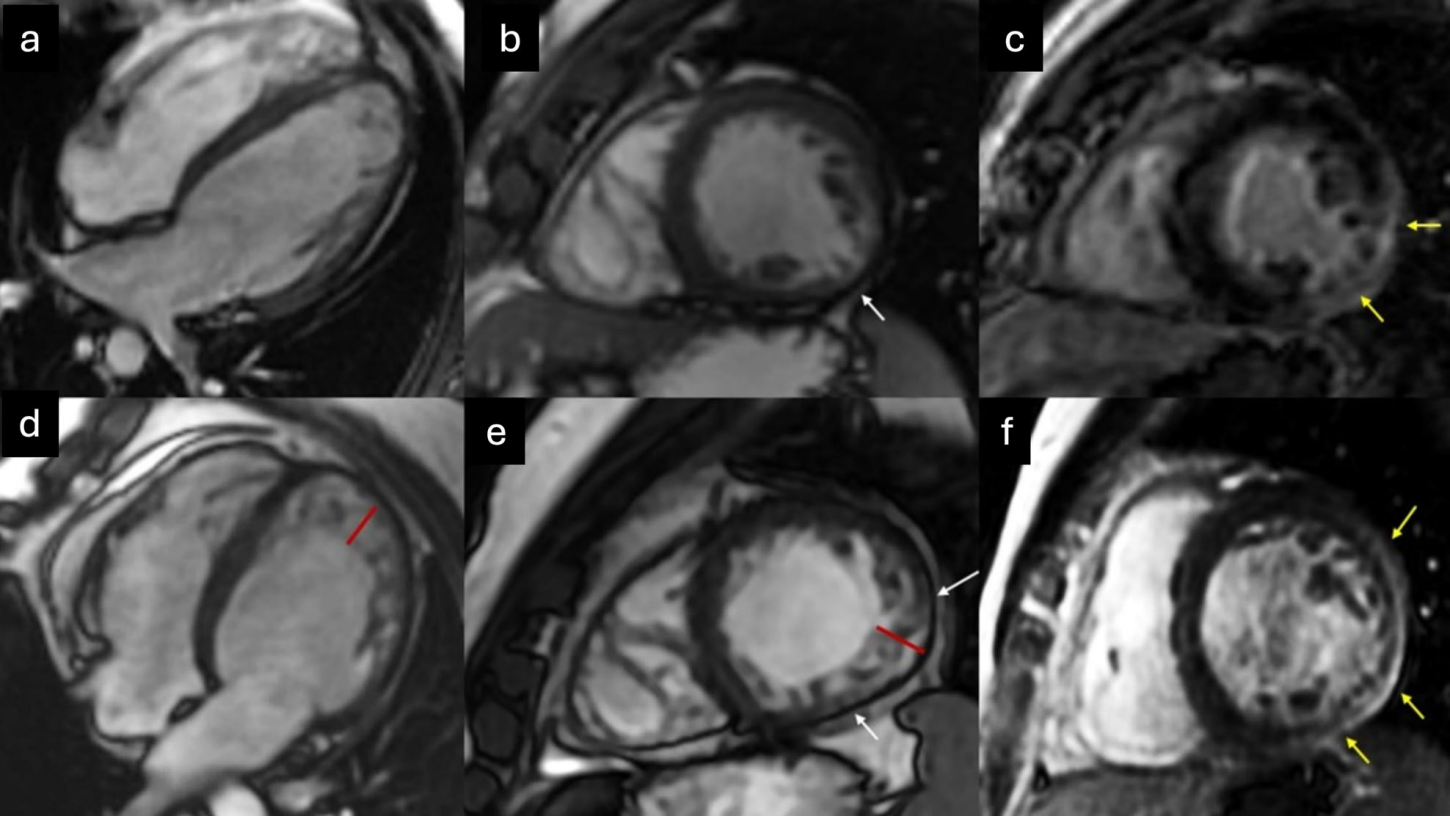
45-year-old male with Friedreich’s ataxia and temporal progression of cardiac involvement. CMR at baseline (**a** SSFP four-chamber cine, **b** SSFP apical short-axis [SA] cine; and **c** LGE apical SA sequence) demonstrates a slightly dilated left ventricle (LV) without ETs and with myocardial thinning (**b**, arrow) of the inferolateral segment. Epicardial LGE is also seen (**c**, arrows). Follow-up CMR 3 years later (**d** SSFP four-chamber cine; **e** SSFP apical SA cine; and **f** LGE apical SA sequence) shows a more dilated LV with ET (red lines in **d**, **e**), more extensive myocardial thinning (**e**, white arrows), and more epicardial LGE (**f**, yellow arrows)

An association has been described between myocardial ET and a wide spectrum of neuromuscular diseases, including Becker and Duchenne muscular dystrophies, myotonic dystrophy, Barth syndrome, and various mitochondrial syndromes. These patients exhibit significant genotypic and phenotypic heterogeneity, although an excess mortality rate has been observed globally, often attributed to conduction system disorders, respiratory muscle dysfunction, increased prevalence of autonomic cardiovascular dysfunction, and reduced mobility. However, the causal relationship between the genetic defect responsible for neuromuscular involvement and the coexistence of ET remains poorly understood [65–67]. Cardiac remodeling associated with progressive myocardial thinning and fibrosis in advanced disease may lead to the appearance of ET [68]. An illustrative case of progressive myocardial involvement with ET in a patient with neuromuscular disease is shown in Fig. 4.

ET has also been linked to various chronic hematologic disorders and hemoglobinopathies, justified as an adaptive response to increased cardiac preload secondary to the hematologic disease. A higher prevalence of ET has been described in patients with β-thalassemia or sickle cell anemia, in most cases without ventricular dysfunction suggestive of an underlying cardiomyopathy [69].

Additionally, chemotherapy used to treat these hematologic disorders has also been associated with the presence of ET, likely secondary to myocardial damage caused by the drug [70].

Several studies have also linked ET to chronic kidney disease, sometimes with findings suggestive of cardiomyopathy, although a clear causal relationship between the two has not yet been established [71]. Given the increased preload in chronic renal failure, ET could once again represent an adaptive response of the myocardium [6].

## ET in the pediatric population

**Fig. 5:**
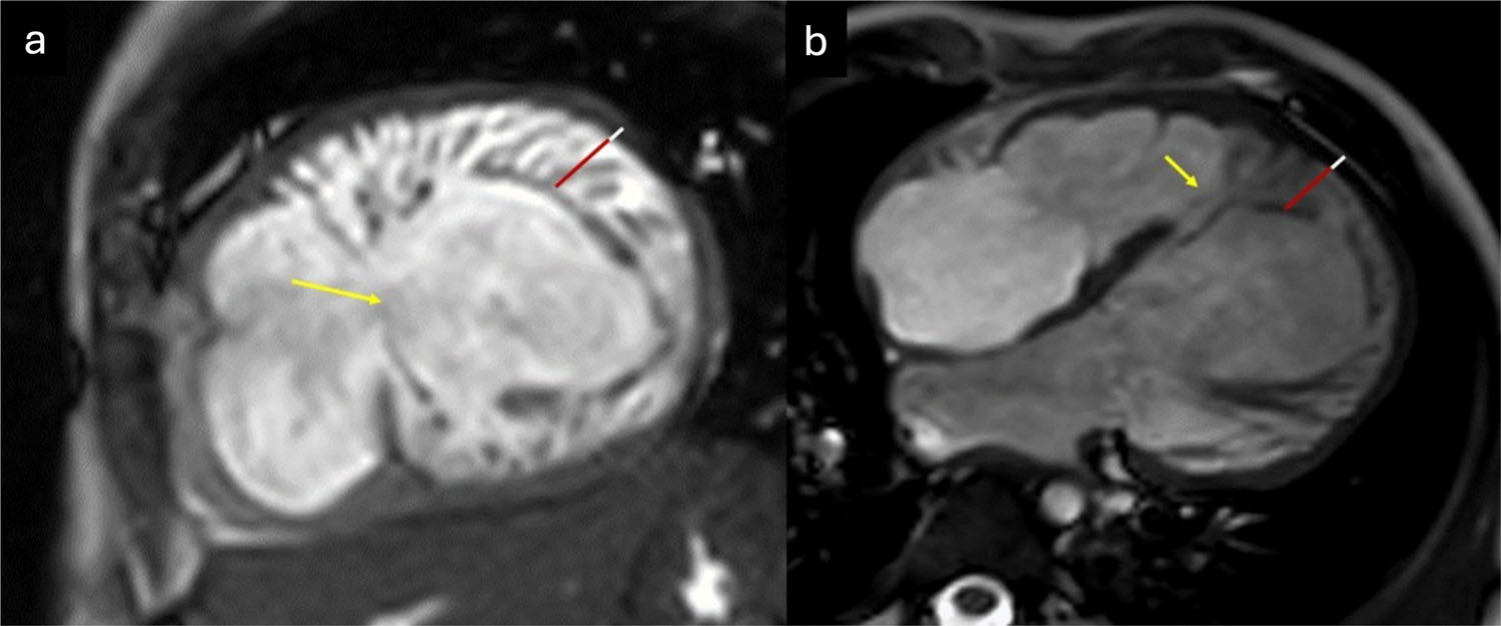
A 21-month-old patient with congenital heart disease (muscular interventricular septal defect [VSD]). CMR SSFP cine images in the short-axis (**a**) and four-chamber (**b**) views show a large interventricular communication (yellow arrows) corresponding to a muscular VSD, associated with diffuse ETs (red lines). The ratio of trabeculated to compact myocardium (white line) exceeds 2.3

As in adults, children can also exhibit morphological criteria of ET on imaging studies. In the absence of dilation or systolic dysfunction, this ET can likewise be considered a phenotypic trait that is not necessarily associated with a cardiomyopathy [72]. However, in children, there is a relatively frequent association with congenital heart disease and various genetic variants associated with cardiomyopathies, neuromuscular disorders, inborn errors of metabolism, and other syndromic conditions. An example of diffuse ET associated with congenital heart disease in a pediatric patient is presented in Fig. 5.

These genetic variants do not seem to predict the risk of developing MACE, while systolic function has been identified as the main independent factor in children [73].

Moreover, the definition of trabeculation thresholds is dependent on age and body size, and physiologic ET in infants and children may represent a transient finding that subsequently normalizes [72].

Due to the limited scientific evidence compared to adults regarding the clinical implications of ET, uncertainties arise that complicate the management of children with this imaging feature [6]. There are no standardized guidelines for follow-up, the need for genetic testing, or the initiation of antiplatelet therapy. Additionally, clear recommendations on the feasibility of engaging in sports activities in this context are lacking [56].

## Complications related to myocardiopathy with ET

Classically, systolic dysfunction was considered the most common complication of LVNC in both adult and pediatric patients, occurring in more than 50% of cases. The progression of dysfunction could lead to major adverse events such as arrhythmias, embolism, or even sudden cardiac death [28, 73, 74].

Regarding systolic dysfunction, it was postulated that myocardial trabeculae caused a hemodynamic disadvantage compared to compact myocardium, which justified the association between ET and heart failure. However, this hypothesis has not been confirmed, and recent studies have found no correlation between the distribution and extent of trabeculation and myocardial function [6].

On the other hand, although the development of re-entrant tachycardias has been linked to the presence of ET, there is insufficient evidence to demonstrate that these individuals have a higher risk of arrhythmias due to a more complex myocardial architecture [75]. Most arrhythmic foci in these patients originate from established areas of fibrosis or regions with less trabeculation, such as the LV outflow tract [76].

Finally, there is also no strong scientific evidence to support that thromboembolic events are more frequent in the presence of ET. Although a potential association between LV ET and intracavitary thrombus formation has been hypothesized, this relationship remains unproven. The presence of deep intertrabecular recesses has been proposed to promote blood stasis and predispose to mural thrombus, particularly in patients with systolic dysfunction, atrial fibrillation, or additional risk factors for thromboembolism. However, large population-based cohort studies have not demonstrated an increased risk of major adverse cardiovascular or thromboembolic events in individuals with ET [77–79]. Therefore, anticoagulation should be considered in the presence of conventional indications such as reduced ejection fraction or previous embolic events.

Overall, although LVNC was initially linked to cardiovascular complications, there is currently no clear evidence that these are directly associated with isolated ET; and they likely reflect the underlying cardiomyopathy.

## **Differential diagnosis**

ET can be misinterpreted as other conditions, especially on initial US, in the context of insufficient expertise, or with technically suboptimal studies. The differential diagnosis of ET can be challenging and has important implications for patient management. The principal differential diagnoses of ET are outlined in Table 4.

**Table 4:** Main differential diagnoses of ETs

Diagnosis	Imaging findings	Clue to distinguish from ET
HCM	• LV wall thickness ≥ 16 mm, • Diastolic wall thickness/volume ratio > 0.15 • Midmyocardial patchy LGE may be seen	• HCM often coexists with ET, making it essential to accurately assess and quantify the thickness of both the compacted and trabeculated myocardium
False tendons	• Endocavitary fibrous band-like structures, that connect the interventricular septum and the papillary muscles, or the ventricular free wall	• Try to follow the continuation between LV walls • False tendons do not attach to the mitral valve
Crypts	• Myocardial recesses oriented perpendicularly to the endomyocardial surface, penetrating more than 50% of the wall thickness, ending to narrow or occlude in systole, and without local hypokinesia or dyskinesia	• Typically disappear or occlude during the systolic phase • Mainly found in the basal inferior septum • Can be identified in the LGE sequences as hyperintense bands, due to Gd endomyocardial deposition
Papillary muscles variations	• Accessory papillary muscles are identified as apical-basal muscle bundle or muscle strands that originates from the LV apex and extends to the basal myocardium without attaching to the mitral leaflet • In the anteroapical displacement, the base of the papillary muscle is positioned more anteriorly and extends more distally	• Frequently observed in individuals with HCM (“soft findings”) • Anteroapical displacement most commonly affects the AL muscle • Orientation of the cardiac planes is of utmost importance to determine the origin and insertion of the papillary muscles and to differentiate them from ET
EMF	• The hallmark morphological characteristic of EMF is the obliteration of the left or right ventricular apex, accompanied by enlargement of the corresponding atrium • The characteristic LGE pattern is subendocardial, not following coronary territories	• A ‘double V’ sign at the ventricular apex is characterized by a three-layered appearance consisting of normal myocardium, a thickened and enhanced endomyocardium, and an overlying thrombus, with or without calcifications
cc-TGA	• There is both atrioventricular and ventriculo-arterial discordance • The most common configuration: morphologic RV lies on the left and supports systemic circulation, with the aorta positioned anterior and to the left of the pulmonary artery • The morphologically RV has a higher trabecular density	• The position of the aortic origin (located anterior and to the left of the pulmonary artery), together with other typical morphologic features of the RV, such as the presence of a muscular infundibulum, will aid in the diagnosis
Saw-tooth cardiomyopathy	• Inward myocardial projections resembling saw-tooth patterns, extending from the lateral walls toward the LV cavity	• Projections of compact myocardium extending into the ventricular cavity, with a thickness greater than that of the typical trabeculations seen in ET, without associated thinning of the compacted layer

*cc-TGA* congenitally corrected transposition of the great arteries, *AL* anterolateral, *EMF* endomyocardial fibrosis, *ET* excessive trabeculations, *HCM* hypertrophic cardiomyopathy, *LGE* late gadolinium enhancement, *LV* left ventricle, *RV* right ventricle

**Fig. 6:**
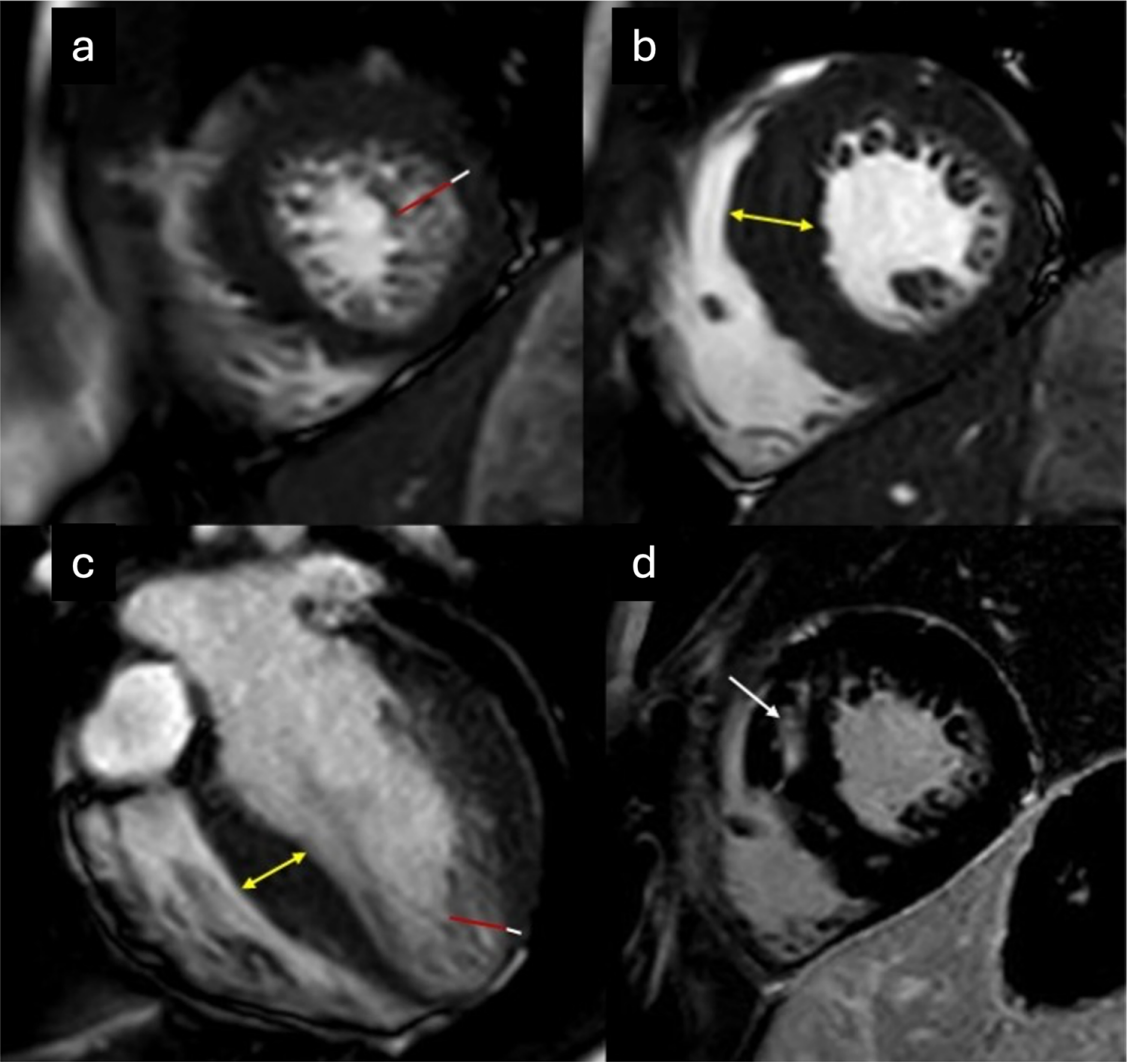
Intercurrent features of HCM and ETs with CMR (**a** SSFP cine short-axis (SA) apical view showing ET [white and red lines]; **b** SSFP cine SA view demonstrating hypertrophy of the mid-septum [double-headed arrow]; **c** SSFP cine four-chamber view showing asymmetric septal hypertrophy [double-headed arrow] with apical ET [white and red lines]; and (**d**) phase-sensitive inversion recovery [PSIR] LGE SA view showing scattered intramyocardial fibrosis [arrow])

Conditions associated with LV wall hypertrophy, particularly hypertrophic cardiomyopathy (HCM), are often mistaken for ET, especially in US [80]. Identifying trabeculations in the apical region of the LV using US can be challenging and may lead to misinterpretation, particularly in the context of HCM. Moreover, ET is commonly observed in HCM and was previously categorized under mixed-phenotype cardiomyopathies. Intermixed features of HCM and ET are illustrated in Fig. 6.

Interestingly, recent studies have suggested that the fractal dimension of apical trabeculation in the LV may serve as an independent predictor of both primary and secondary clinical outcomes in patients with HCM [81]. Cine sequences on CMR provide superior spatial resolution, enabling clear differentiation between compact myocardium, trabeculae, and intertrabecular recesses [27].

**Fig. 7:**
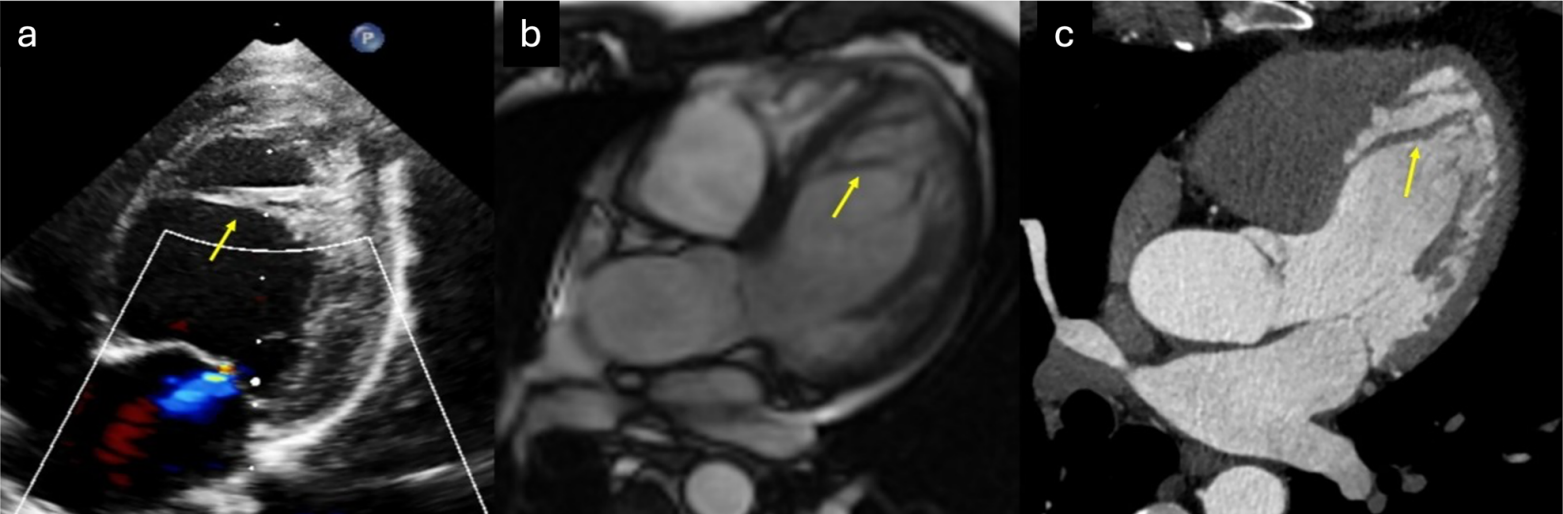
False tendons (aberrant bands) in two different patients studied with US, CMR, and cardiac CT, ((**a**) US in a newborn shows a dilated LV with a large aberrant muscular band (arrow) connecting the interventricular septum to the lateral free wall, which may be mistaken for ET, (**b**) SSFP cine CMR three-chamber view, and (**c**) cardiac CT) depict large aberrant muscular bands (arrows) mimicking apical ET. Both CMR and cardiac CT, through multiplanar evaluation and high contrast resolution, allow accurate definition of the anatomy and course of the aberrant bands

False tendons or aberrant bands are fibrous bands that extend across the LV cavity, connecting the septum, papillary muscles, or ventricular free wall, but they do not attach to the mitral valve [82]. Their functional role remains poorly understood. These structures are present in nearly half of human hearts examined post-mortem. Some theories propose that false tendons help limit LV remodeling by providing structural support, and that they might play a role in mitigating functional mitral regurgitation by stabilizing papillary muscle positioning during LV dilation, but none of these have been demonstrated. However, false tendons may also have adverse effects, as they have been linked to the development of membranes in cases of subaortic stenosis [83]. When prominent, these false tendons can be mistaken for ET, especially when evaluated using US. Examples of false tendons mimicking ET are shown in Fig. 7.

**Fig. 8:**
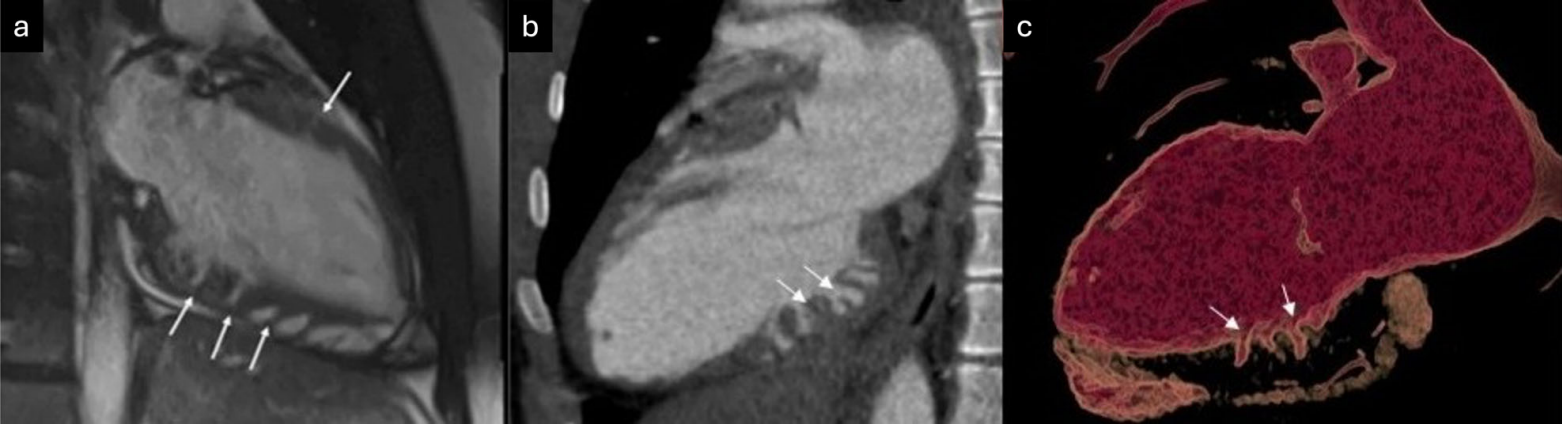
Myocardial invaginations perpendicular to the endomyocardial surface, predominantly in the inferoseptal wall of the LV, corresponding to myocardial crypts (arrows). These can be identified both on CMR (**a** SSFP cine two-chamber view) and CT (**b** two-chamber multiplanar reconstruction, **c** two-chamber volume rendering)

Myocardial clefts or crypts are narrow, deep invaginations in the myocardium, identified as muscle recesses oriented perpendicularly to the endomyocardial surface, typically V- or U-shaped, and penetrating more than 50% of the myocardial wall thickness, ending to narrow or occlude in systole and without local hypokinesia or dyskinesia [84]. They are primarily found in the basal inferior septum and in the LV free wall. While myocardial clefts may be significant in individuals with a high likelihood of HCM, their clinical relevance remains uncertain when detected in isolation, often representing incidental and benign structural variations [85]. When multiple and deeply distributed in the mid-apical region, they may be mistaken for ET. Typical imaging features of myocardial crypts that may be confused with ET are displayed in Fig. 8.

**Fig. 9:**
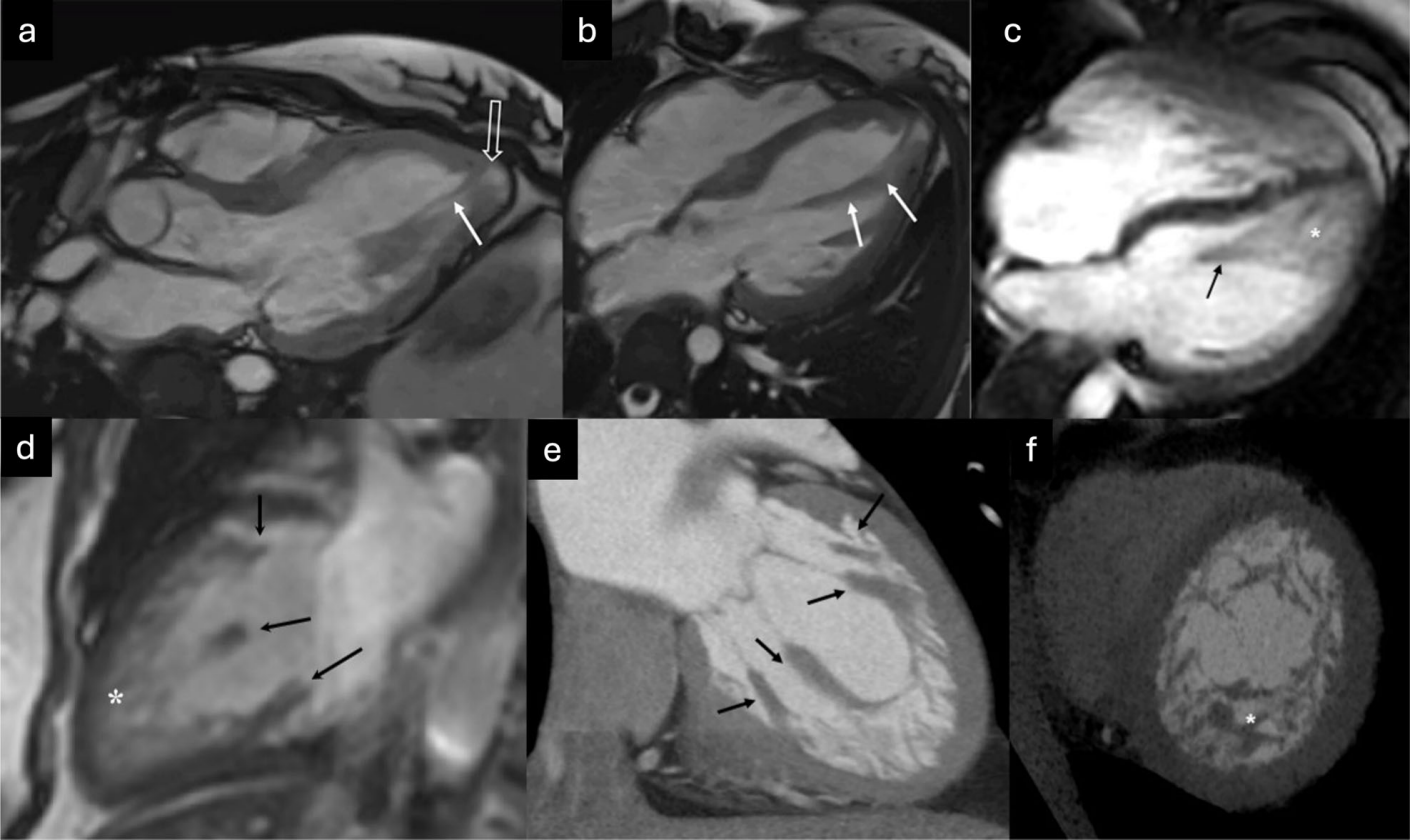
Papillary muscle anatomic variants. CMR (**a** SSFP three-chamber cine; **b** SSFP four-chamber cine; and **c** SSFP four-chamber cine) shows apical insertion of the posteromedial papillary muscle (solid arrows in **a**, **b**) in a patient with HCM. Note the small apical aneurysm of the LV (hollow arrow in **a**). **c** Apical insertion of the posteromedial papillary muscle in another patient (black arrow) generates apical ETs (asterisk). Accessory papillary muscle in a different patient is shown with CMR (**d** SSFP two-chamber view) and CT (**e** two-chamber view, **f** short-axis minimum-intensity projection) (arrows), associated with ET (asterisk)

LV papillary muscles are small myocardial structures essential for the function of both the mitral valve and the LV. They are generally classified into two groups: the anterolateral and posteromedial papillary muscles. Notably, papillary muscles exhibit significant morphological variability, for instance, accessory papillary muscles and anteroapical displacement [86]. Accessory papillary muscles are identified on CMR or CT as apical-basal muscle bundles or muscle strands that originate from the LV apex and extend to the basal myocardium without attaching to the mitral leaflet. This variation is frequently observed in individuals with HCM, occurring in 63% of patients and 60% of gene-positive family members, compared to only 10% of controls [87]. In anteroapical displacement of the papillary muscles, the base of the papillary muscle is positioned more anteriorly relative to the ventricular septum and extends more apically. Normally, it can be accurately visualized in cine CMR images using 2- or 4-chamber views. This configuration is significantly more prevalent in patients with HCM compared to healthy controls (77% vs 17%) and most commonly affects the anterolateral muscle. Accessory papillary muscle, anteroapical displacement variations, and bifid papillary muscles with multiple bellies can sometimes be misinterpreted as ET in imaging studies [88]. Representative CMR and CT images of papillary muscle variants that can resemble ET are shown in Fig. 9.

**Fig. 10:**
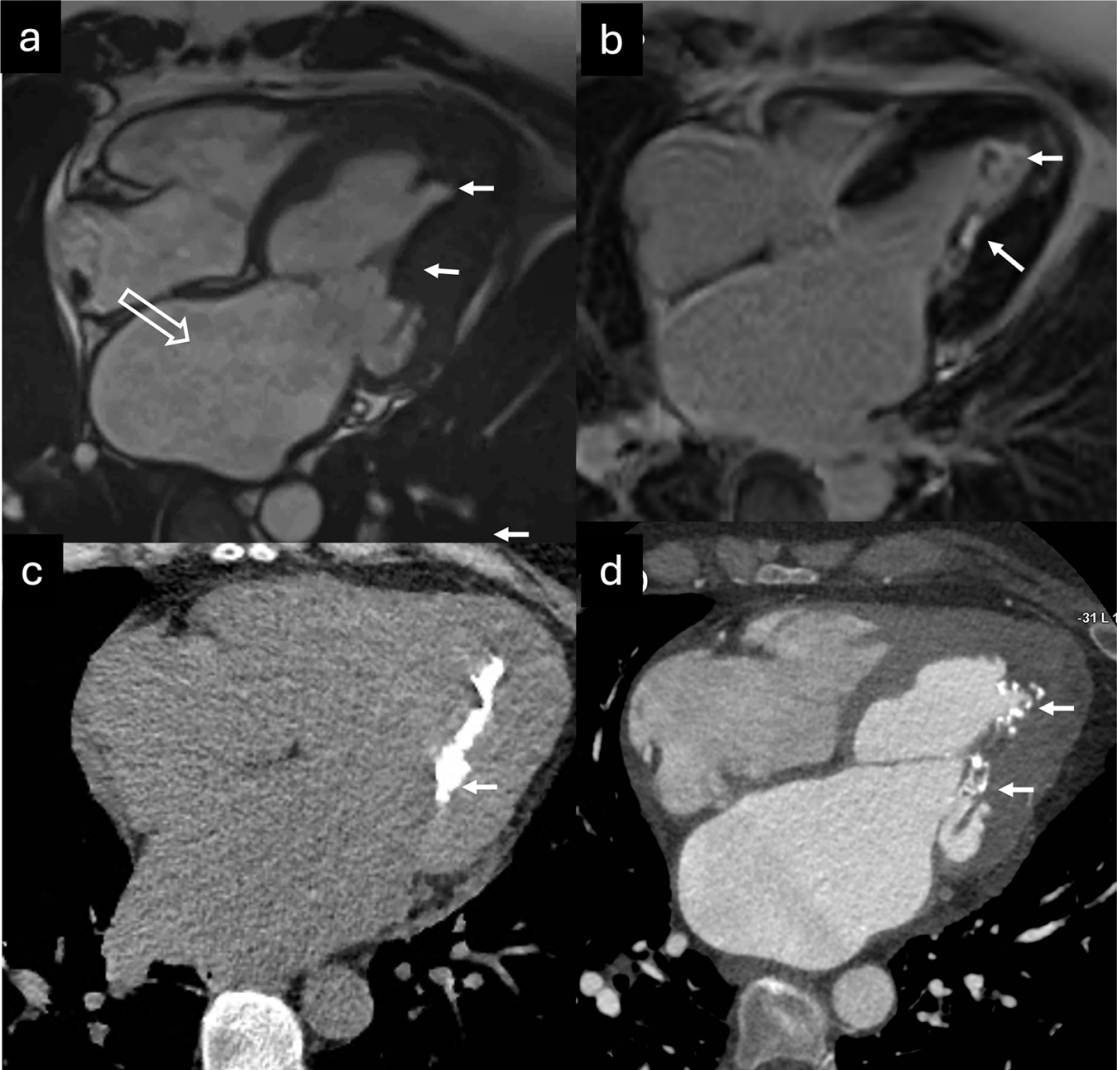
A 52-year-old patient with chronic EMF. CMR (**a** SSFP cine four-chamber view and **b** LGE image) and cardiac CT (**c** noncontrast and **d** contrast-enhanced images) show partial obliteration of the LV cavity by fibrosis extending to the papillary muscles and an intracavitary thrombus (solid arrows), which may mimic ETs. Note the associated left atrial dilation (hollow arrow in **a**) and partial calcification of the intracavitary thrombus and fibrotic myocardium (arrows in **c**, **d**)

Endomyocardial fibrosis (EMF) is a rare form of restrictive cardiomyopathy predominantly seen in tropical and subtropical regions. Its pathogenesis remains unclear, but it is thought to result from a combination of factors. Parasitic infections, socioeconomic disadvantage, malnutrition, and genetic predisposition are believed to contribute to inflammation and immune modulation, promoting a profibrotic state. This process ultimately leads to EMF, characterized by ventricular apical obliteration, atrial enlargement, and atrioventricular valve regurgitation. The hallmark morphological characteristic of EMF is the obliteration of the LV or RV apex, accompanied by enlargement of the corresponding atrium [89]. This feature is readily detectable using cine SSFP imaging. The characteristic LGE pattern in EMF is subendocardial, not following specific coronary territories, primarily affecting the apex. Notably, the outflow tract orifices are typically preserved. The “double V” sign at the ventricular apex, which presents a three-layered structure comprising normal myocardium, thickened enhanced endomyocardium, and an overlying thrombus, with or without calcifications, has been described [90]. In some scenarios, EMF may pose a diagnostic dilemma with ET, especially when only US is available. The characteristic imaging appearance of EMF that can mimic ET is depicted in Fig. 10.

**Fig. 11:**
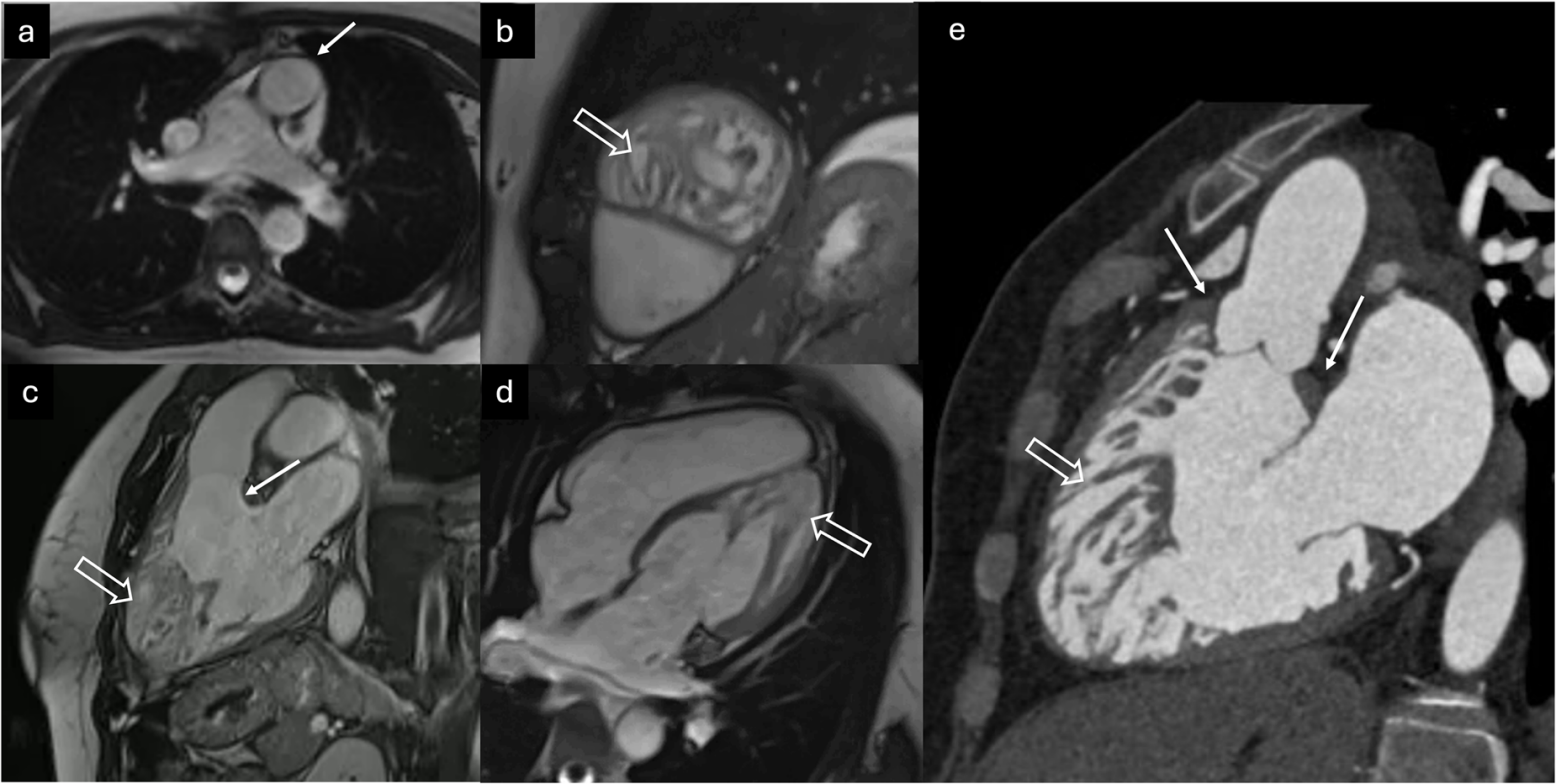
Unrecognized cc-TGA in a 23-year-old patient evaluated for ECG abnormalities and referred for CMR due to ET detected on US. CMR (**a**–**d**) and cardiac CT (**e**) demonstrate atrioventricular and ventriculoarterial discordance, with an anterior and leftward position of the aorta relative to the pulmonary trunk (arrow in **a**, axial localizer SSFP image); a left-sided morphologic RV with marked ET (hollow arrows in **b**–**d**, cine SSFP sequences in short-axis, three-chamber, and four-chamber views, respectively; hollow arrow in (**e**), contrast CT three-chamber reconstruction); and a muscular band between the atrioventricular and semilunar valves (solid arrows in **c**, **e**)

Congenitally corrected transposition of the great arteries (cc-TGA) is a rare congenital heart defect, accounting for less than 1% of cases, characterized by both atrioventricular and ventriculo-arterial discordance that results in physiologically corrected circulation. In the most common configuration, the morphologic RV lies on the left and supports systemic circulation, with the aorta positioned anterior and to the left of the pulmonary artery. Although isolated cc-TGA may remain asymptomatic for decades, most patients present associated anomalies such as ventricular septal defect, pulmonary stenosis or subpulmonic obstruction, and tricuspid valve dysplasia or Ebstein-like malformation, which determine symptoms and prognosis [91]. Since the morphologic RV is positioned on the left and naturally exhibits greater trabecular density, unrecognized cc-TGA may be misinterpreted as ET of a misidentified LV. Characteristic imaging features of cc-TGA that may mimic ET are illustrated in Fig. 11.

**Fig. 12:**
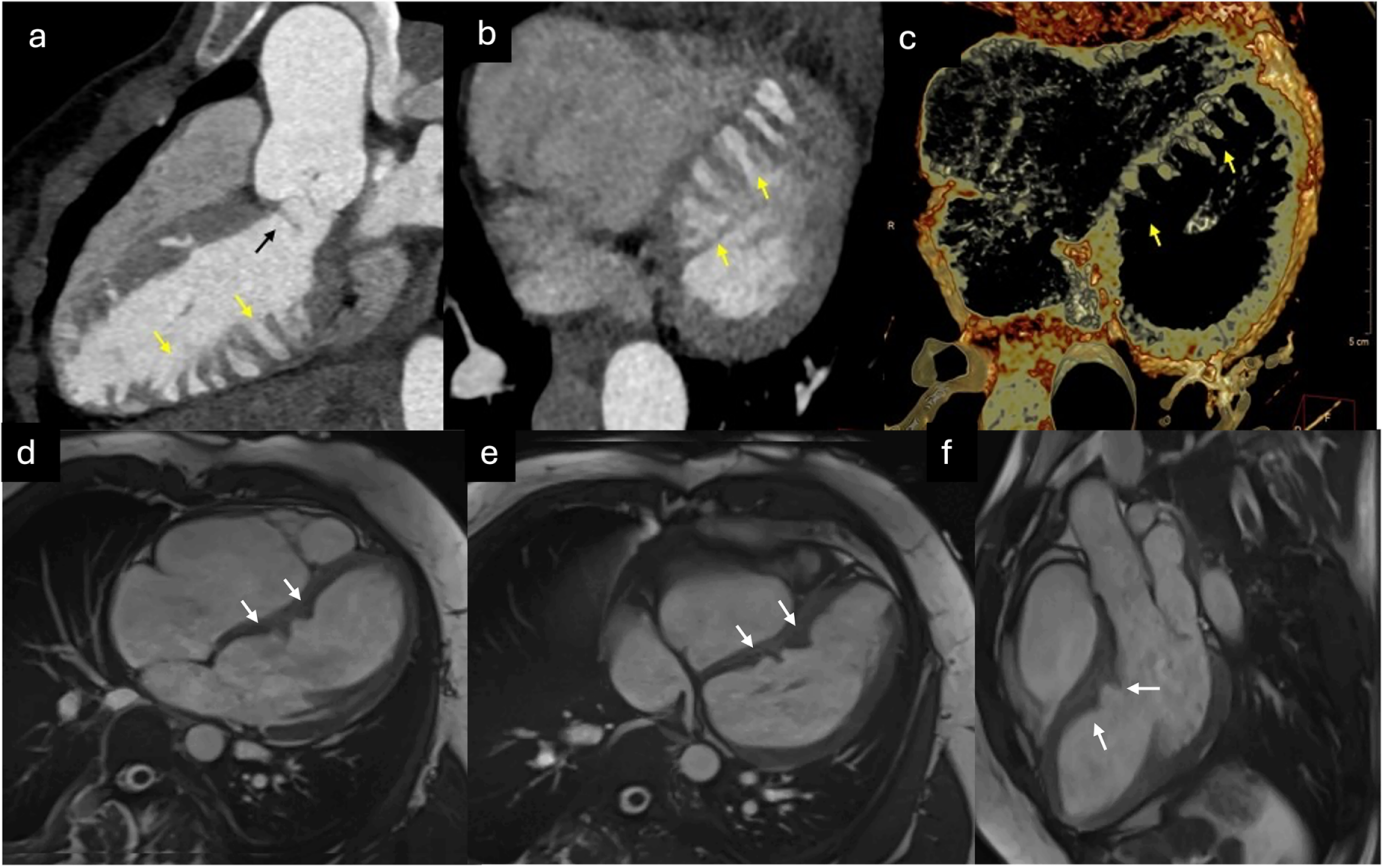
Saw-tooth cardiomyopathy in two different patients with two different patterns. Cardiac CT (**a** sagittal oblique image, **b** axial oblique plane, and **c** volume-rendered axial oblique image) in a 13-year-old patient with aortic coarctation, left isomerism, and subaortic membrane (**a** black arrow) incidentally demonstrates inward myocardial projections (yellow arrows) resembling a saw-tooth shape, extending from the septum to the lateral wall, suggestive of saw-tooth cardiomyopathy (cript-like pattern). CMR (SSFP four and three-chamber view cine images in **d**–**f**, respectively) in a 42-year-old patient submitted for unspecific ECG abnormalities, with myocardial projections from the anteroseptal wall of the LV to the cavity with the saw-tooth appearance (arrows) (false tendon-like pattern)

Lastly, saw-tooth cardiomyopathy is an extremely rare condition that was initially misclassified as a variant of LVNC, characterized by inward myocardial projections resembling saw-tooth patterns, extending from the lateral walls toward the LV cavity [92]. Although it may resemble ventricular trabeculations, the presence of septal dysplasia and muscular bridges between the inferior and lateral walls supports the correct diagnosis. Examples of saw-tooth cardiomyopathy with distinct imaging patterns are presented in Fig. 12.

## Conclusions

LV ET is now regarded as a morphological trait that may lack pathological significance in individuals without other cardiac abnormalities. Its classification as a distinct cardiomyopathy—formerly known as LVNC—is no longer recommended. When ET is present alongside features suggestive of cardiomyopathy, accurate diagnosis becomes essential, with multimodality imaging playing a key role. Beyond morphological assessment, it is critical to incorporate functional parameters (such as ventricular size and systolic function) and tissue characterization (mainly through LGE on CMR) to ensure a comprehensive evaluation and enable appropriate prognostic stratification. ET may also occur in the context of hemodynamic overload or be associated with systemic conditions, including neuromuscular disorders. In pediatric patients, its interpretation may differ, as scientific evidence remains limited, and it is often found in conjunction with other abnormalities—most commonly congenital heart defects. Lastly, it is important to recognize that several other cardiac entities can mimic ET, highlighting the need for a thorough differential diagnosis.

## Data Availability

No new data were generated or analyzed in this study. Data sharing is therefore not applicable.

## Abbreviations

ARVC: Arrhythmogenic right ventricular cardiomyopathy
CMR: Cardiac magnetic resonance imaging
DCM: Dilated cardiomyopathy
ET: Excessive trabeculation
HCM: Hypertrophic cardiomyopathy
LGE: Late gadolinium enhancement
LV: Left ventricle
LVNC: Left ventricular “non-compaction”
RV: Right ventricle
SA: Short axis
SSFP: Steady-state free precession
US: Echocardiography
