# Correction: How should we interpret excessive left ventricular trabeculation? Update on controversies from the cardiac imaging perspective

**DOI:** 10.1186/s13244-026-02325-1

**Published:** 2026-06-09

**Authors:** Roque Oca Pernas, Nerea Hormaza Aguirre, Marcos Mestas Nuñez, Ana Capelastegui Alber, María Navallas Irujo, Ana García Durán, Alex Pérez Casares, Flavio Zuccarino

**Affiliations:** 1 MR Department, OSATEK Deusto, Osakidetza-Bask Health Service, Bilbao, Spain; 2 Radiology Department, Cruces University Hospital, Biocruces Bizkaia, Barakaldo, Spain; 3 Radiology Department, Hospital del Mar University Hospital, Barcelona, Spain; 4 MR Department, OSATEK Galdakao University Hospital, Osakidetza-Bask Health Service, Galdakao, Spain; 5 Radiology Department, Sant Joan de Deu University Hospital, Barcelona, Spain; 6 Cardiology Department, Hospital del Mar University Hospital, Barcelona, Spain; 7 Cardiology Department, Sant Joan de Deu University Hospital, Barcelona, Spain

**Correction to: Insights into Imaging** (2026) 17:129

10.1186/s13244-026-02264-x; Article published online 14 May 2026

In this article, the author’s name Marcos Mestas Nuñez was incorrectly written as Marcos Mestas Nuñes.

The original article has been corrected.

